# *Entropy* 2022 Best Paper Award

**DOI:** 10.3390/e24050724

**Published:** 2022-05-20

**Authors:** Entropy Editorial Office

**Affiliations:** MDPI, St. Alban-Anlage 66, 4052 Basel, Switzerland; entropy@mdpi.com

On behalf of the Editor-in-Chief, Prof. Dr. Kevin H. Knuth, we are pleased to announce the *Entropy* Best Paper Award for 2022. 

Papers published in 2020 were preselected by the *Entropy* Editorial Office based on the number of citations and downloads from the website. The winner nominations were made by a selection committee, which was chaired by the Editor-in-Chief and supported by eighteen Editorial Board Members. The five top-voted papers, in no particular order, have won the 2022 *Entropy* Best Paper Award:

**Thermodynamics in Ecology—An Introductory Review [1]**

**Søren Nors Nielsen, Felix Müller, Joao Carlos Marques, Simone Bastianoni and Sven Erik Jørgensen**

***Entropy* 2020, 22(8), 820; https://doi.org/10.3390/e22080820**


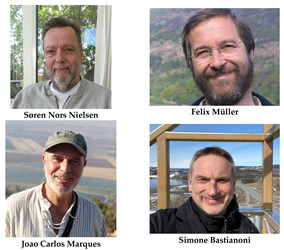



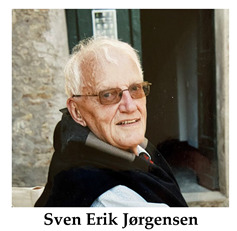


In the early 1980s, there was a wish among ecologists to improve existing ecological models and make them able to mimic the natural processes that occur during ecosystem evolution and development, such as, for instance, the adaptation of species properties as well as shifts in species composition that take place in communities during succession. From the outset, this wish challenged the scientific knowledge regarding the actual causes of the changes observed in various ecosystems that had been gathered through empirical works throughout the century. Most ecologists have thorough knowledge of the phenomenological properties described by E.P. Odum which were deduced through his intensive studies on ecosystems worldwide.

E.P. Odum predicted and described succession to take place through an orderly process, during which, different species were replacing each other, with the whole ecosystem ending up in a homeostatic state in which primary production was balanced with the total respiration of the end state. This state has often been referred to as “the climax society”. The many shifts observed during evolution and sequences of balances—constantly being perfectly matched with inputs and outputs of energy and matter—were seen by many physically oriented ecologists as a demonstration that thermodynamic laws could potentially be applicable to ecosystems and life in general, as previously hypothesized by Lotka and Shrödinger, although ecosystems clearly represent states far from equilibrium and also, presumably, linear dependencies between flows and forces, which was assumed by Onsager.

The many applications demonstrated today and the increasing number of papers published in the area clearly confirm the relevance of the approach, but also, as this review illustrates, this approach has weaknesses as well as strengths. Many of these limitations are clearly revealed when one tries to teach the application of these matters to students—both at the graduate and post-graduate level. This paper began with an attempt to shed ligth on some of the problems emerging, in particular when one is a newcomer to the area of thermodynamics in ecology—which eventually became the title of the paper. We have tried to deal most with the entropy and exergy approaches and the problems in moving such concepts to the far from equilibrium domain. Unfortunately, we left out an important discussion on entropy versus information, determining that this would be an unnecessary complication.

What became clear throughout our studies is that our human society, in a thermodynamic sense, acts as immature ecosystems, and that we thus may learn a lot from studying ecosystem behavior if we want to develop by shaping a sustainable world. Resources considering both energy and matter need to be used in a much more sensible manner, reducing the need for supplies by the introduction of a circular economy, which has to work in close connection with available energy. A fair distribution concentrating on equity can be implemented but will force us to focus much more on the third world, as demonstrated in the book *Flourishing within Limits to Growth*, a publication which several of the authors of this paper also contributed to.

**The Role of Entropy in the Development of Economics [2]**

**Aleksander Jakimowicz**

***Entropy* 2020, 22(4), 452;** **https://doi.org/10.3390/e22040452**


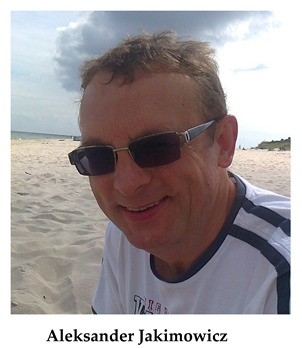


Economics and physics have been feeding into each other for at least 170 years. Their partnership dates back to the first half of the 19th century, when French engineers—somewhat ‘en passant’, as they were working on various technical projects—laid the methodological groundwork for the development of neoclassical economics. The neo-classicists raised the academic status of economics by expanding it with the ideas and mathematical apparatus of energy physics, which then developed into thermodynamics. Thus, the law of equilibrium was transferred from physics to economics, and unfortunately, this was also the origin of the dogma still held by mainstream economics that markets and economies are closed systems that tend toward equilibrium. The reverse relationship is also true, with many examples of economics fueling the development of physics. These include, in particular, power laws, the random walk, and the sensitivity of non-linear systems to initial conditions. Granted, in the case of the last example, we now know that deterministic chaos was discovered in 1890 by Henri Poincaré as he was studying the restricted three-body problem, but chaos became a staple of sciences only after Edward Lorenz’s 1963 work on chaotic dynamics in a non-linear model for atmospheric convection. It is a little-known fact that Lorenz’s finding was preceded by two other descriptions of chaos by economists: in the duopoly and oligopoly models in 1936–1939, as well as in Goodwin’s non-linear business cycle model in 1953.

The concept of entropy crystallized in physics after the observation that friction and dissipation prevented steam engines from converting a large portion of energy into useful work. Rudolf Clausius termed this missing energy ‘entropy’ and presented the first mathematical definition of it in 1854. The same scholar created a beautifully concise formulation of the first two laws of thermodynamics:

1. The energy of the universe is constant.
2. The entropy of the universe tends to a maximum.

Since then, the entropy-based second law of thermodynamics has become an analytical and methodological cornerstone of both physics and economics. The importance of entropy for the growth of economic sciences was emphasized both by the Italian physicist Ettore Majorana in a 1942 paper and by the Polish economist Zygmunt Rawita-Gawroński in a work published in 1958. The impact of thermodynamic entropy on the empirical capacity of economics has been enormous. In fact, it would not be an overstatement to say that entropy finally opened the gateway to the precise formulation of major economic concepts, such as utility or the value of goods and services—concepts without which, modern economics would not exist. In 1971, the Romanian-born American economist Nicholas Georgescu-Roegen stated that thermodynamics is not actually about explaining physical phenomena governed by heat transfer, but about the desire for a deeper understanding of economic processes. This is why he considered thermodynamics to be the physics of economic values, and why he thought the law of entropy is the most economy-like law of physics. The most important aspect of production processes is the conversion of low-entropy factors of production into high-entropy final goods and services. Thus, if any good or service is to be useful, it must have low entropy—their economic value is derived from their utility, and hence from their low entropy.

The main aim of this article is to explore the influence of the general concept of entropy on economics. Entropy as a physical phenomenon expressed in the second law of thermodynamics serves as the starting point for this discussion. The incorporation of entropy into the theory of production has given rise to a new field of research—ecological economics or bioeconomics. Production processes constantly expend and diminish low-entropy resources available to humans, causing the scarcity of goods. Following this observation, the entropy economics paradigm states that irreversibility is a main feature of economic phenomena. Despite the claims of mainstream economics, economic flows do not exclusively drive the circular flow of income and expenditure—they can also be unidirectional. Normal production processes generate waste that firstly needs to be expelled and then offset by an influx of new resources from the environment. Therefore, the circular flow is accompanied by the linear throughput of matter-energy, fed by the constant movement of money, goods, and services, as well as factors of production. Linear throughput refers to the influx of low-entropy natural capital, such as solar energy, mines, wells, fisheries, croplands, and the outflow of high-entropy wastes. The ultimate validation of largely irreversible production processes lies in the fact that they give people joy and satisfaction in life. Production processes are based on the value that life holds for each person. This explains both why people engage in business ventures and what the fundamental purpose of their economic activity is—the survival of humanity as a species.

Thermodynamic entropy is often used as a metaphor in organization and management sciences. There has even been a second thermodynamic law of management, positing that just as entropy always increases in the universe, so it does in organizations. A new field of study has also appeared—thermoeconomics—which applies the analogues of thermodynamic entropy in economics, as best exemplified by money entropy.

Ever since thermodynamic entropy was first defined by Rudolf Clausius, the science of entropy has made huge strides. This not only feeds into natural sciences, but also economics, which has found applications for many forms of entropy. The most interesting examples include Shannon’s information entropy and non-extensive Tsallis entropy, both of which have been great contributions to econometrics. The field has even seen the emergence of a new school—that of non-extensive cross-entropy econometrics, which serves as a valuable supplement to traditional econometrics by incorporating phenomena based on power-law probability distribution and supports econometric model estimation for non-ergodic, ill-behaved inverse problems. Many applications harness entropy-maximizing probability distribution as a method of statistical inference.

Markets and economies possess identifiable dissipative structures with steady states that are far from equilibrium. These systems exhibit non-extensive entropy, which is not additive. As such, entropy in an economic system is a sum of the entropy production in the system itself and the exchange of entropy with the environment. Negative entropy derived from the environment can reduce entropy production in the same system. If disrupted, such systems do not always reach an equilibrium of maximum entropy. As noted by Ilya Prigogine, states which are far from equilibrium produce ordered structures based on long-range correlations. Phenomena occurring therein have long-term memory. In such systems, interactions between internal and external elements cause energy dissipation. The discovery of dissipative structures in actual objects helped establish the scientific basis for the study of complex adaptive systems.

If entropy and information are closely interlinked, then—as noted by Murray Gell-Mann—ignorance may also be measured in terms of entropy. This means that even though information and ignorance are polar opposites, they can be measured with the same metric. In this view, entropy can be considered the sum of the average ignorance for a given microstate of a system within a macrostate, and of the ignorance describing the macrostate itself. The generalized measure of ignorance presented in the article paves the way for a formulation of complex systems functioning at the edge of chaos—a subject of complexity economics. There have been many types of “edges of chaos” identified in economic systems, including chaotic attractors and repellers, catastrophes of complexity, the coexistence of attractors, sensitive dependence on parameters, final state sensitivity, the effects of fractal basin boundaries, and chaotic saddles.

Entropy economics has also laid the groundwork for new avenues of economic research, such as econophysics, complexity economics, and quantum economics. Econophysics has illustrated the limited veracity of the efficient market hypothesis, whereas complexity economics has demonstrated that markets and economies are most efficient at the edge of chaos. Finally, quantum economics has shown that the value of a good is usually indeterminate, so that a price can only be determined after a transaction, with money used as a measurement device. Now, as before, economics must turn to physics for new methods—but it needs to be the physics of the 21st century, not the 19th, that it draws from.

**Large Deviations for Continuous Time Random Walks [3]**

**Wanli Wang, Eli Barkai and Stanislav Burov**

***Entropy* 2020, 22(6), 697;** **https://doi.org/10.3390/e22060697**


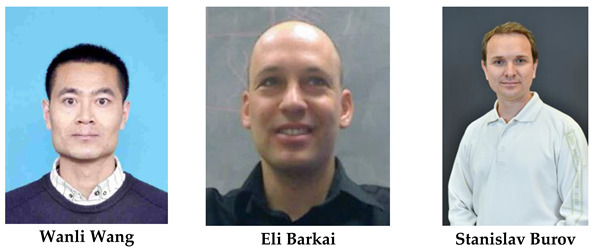


Normal diffusion, for example, Brownian motion, is a Gaussian process described by the central limit theorem in the long-time limit. However, exponential decays of the positional probability density function of packets of spreading random walkers were observed in numerous situations that include glasses, live cells, and bacteria suspensions. Exponential tails, in turn, are related to so-called Laplace distribution; thus, our work is related to a clash between two schools of thought associated with Laplace and Gauss. Here, we promote the basic theory describing this important phenomenon based on the well-known continuous-time random walk model. In some sense, our work extends the central limit theorem to also include the widely observed Laplace-like tails of the spreading packet of particles, using a framework called large deviation theory. As our theory shows, the exponential decay of the packet is universal, and hence, the theory discussed here is proven to be very important in many contexts.

One observation in the field is that in many cases, the spreading of the packet of particles is not described by simple Brownian motion, as expected from Einstein’s theory of diffusion. When the averaged number of jumps recorded under a microscope (or in simulations) within an observation time is not large, one would naively expect that this would imply the non-existence of universal statistical laws. In this paper, we show that such exponential behavior is generally valid in a large class of problems of transport in random media based on the well-known continuous-time random walk model. Under mild conditions in which the microscopic jump lengths’ distribution decays exponentially or faster, and the distribution of the waiting times is analytical for short waiting times, the spreading of particles follows an exponential decay pattern at large distances, with a logarithmic correction. The theory reveals that rare fluctuations describing the large displacement comes from a large number of renewals, which is totally different from the single big jump principle. Here, we further show how the anti-bunching of jump events reduces the effect, while bunching and intermittency enhances it. We employ exact solutions of the continuous-time random walk model to test the large deviation theory.

**Thermodynamics at Very Long Time and Space Scales [4]**

**Bjarne Andresen and Christopher Essex**

***Entropy*** **2020, 22(10), 1090; https://doi.org/10.3390/e22101090**


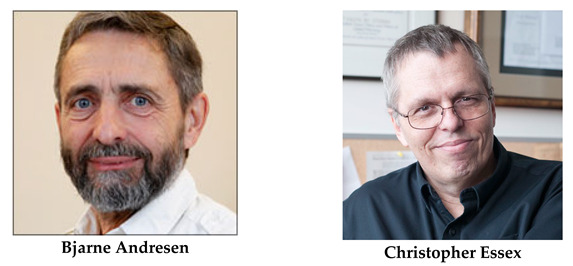


Any direct observation of the natural world is limited by the native time and length scales of an observer’s instruments. At very short times and lengths we can neither establish ‘before and after’ nor distinguish individual objects. Similarly, changes over very long times are not observed directly, neither are changes very large in space. 

Originally humans envisioned scales of minutes and meters, give or take an order of magnitude or two. Our understanding was naturally biased toward human scales and concepts. Notions like volume, pressure, and temperature of continuous media matched our experience well. However, over the past 150 years we found ways to reveal new regimes existing on molecular scales, involving nanometers and nanoseconds—far below human perception. This led to atomic physics which required completely new conceptualizations of nature, while also requiring us to contend with how human scale properties emerged from that new unseen regime, e.g., temperature and irreversibility. Within that regime there were more distinct regimes: nuclear, subnuclear etc., each largely invisible to the regimes containing them. 

Does this ladder of regimes and scales end with human scales? To address this, we turn this concept around and ask what we might expect of a very large and slow regime where humans were “atomic”—too fast and small to see. We call this outlook “slow time”. From this standpoint we explore which laboratory concepts still apply for “slow time” and which new ones may emerge. E.g., we find that temperature as we know it cannot exist and new hidden properties emerge that can be addressed in the spirit of entropy, but for exterior (long time) scales instead of interior (human time) ones. 

Just as finite-time thermodynamics developed from the small additional constraint of a finite process duration, we add a small new condition, the very long length and time scales that result in a loss of spatial and temporal resolution, and again look for new structure. 

As a simple illustration of our ideas, we photographed the busy traffic with lots of students, cars, trucks, and busses moving about at an intersection on the University of Western Ontario campus in two different ways. One picture was a normal photograph taken at 1/100 s with all these objects clearly visible. Another picture of exactly the same scenery was exposed for 10 min. In the latter, no moving objects are seen anymore, except for a few very faint shadows, and the red-yellow-green traffic lights are all lit at the same time, on average. It is a “ghost town”. These pictures illustrate the presence of structure (or lack thereof) appropriate to the different timescales. 

Curious to see these pictures and more? Read the paper. 

**Geometric Optimisation of Quantum Thermodynamic Processes [5]**

**Paolo Abiuso, Harry J. D. Miller, Martí Perarnau-Llobet and Matteo Scandi**

***Entropy*** **2020, 22(10), 1076; https://doi.org/10.3390/e22101076**


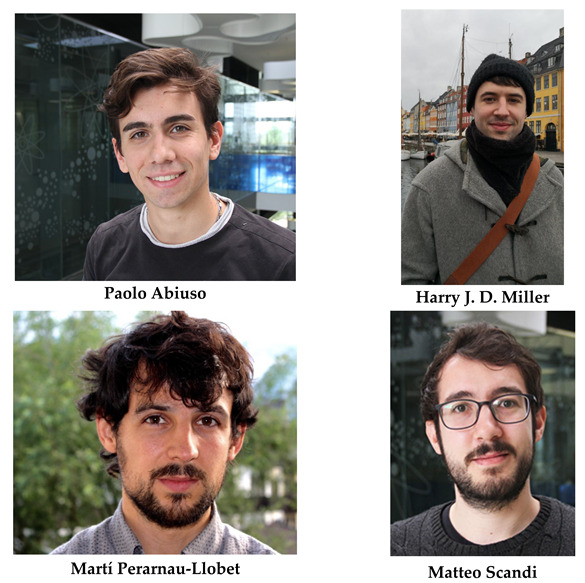


Sadi Carnot, the “father of thermodynamics”, was interested in improving the efficiency of steam engines, to be used in the Napoleonic wars. As such, the quest for the performance optimization of thermal machines is as old as the whole field of thermodynamics. The typical goal is to improve the machine *efficiency* or the machine *power*, which are defined according to the specific task (engine, refrigerator, etc.). Generally speaking, dissipation is detrimental to both these desiderata, and optimal strategies are also minimally dissipating according to the given constraints. The optimization is to be performed over possible cycles that can be performed by modulating the control parameter of the devices at hand. In general, such optimization is a challenging task, as in the most general case, one would need to understand the non-equilibrium dynamics and then make use of variational calculus and optimal control theory. Universal results are only known for the case of quasistatic, reversible thermodynamics, which is of little use when one is interested in finite-time operations.

Notably, in a regime where the control on a machine is operated in a slow but finite time, universal results can be partially recovered, due to time-scaling considerations and a peculiar geometrical structure arising on the manifold of thermal states. In fact, in such a close-to-equilibrium regime, a metric can be defined over a set of thermal states, which quantifies the dissipative losses via its integrated length, also known as *thermodynamic length.* Interestingly, these considerations do not only apply to macroscopic engines, but also to mesoscopic and quantum engines and refrigerators. However, the presence of non-trivial relaxation dynamics and (possibly) quantum coherence makes the thermodynamic geometric structure more complex and has been a subject of intense research in recent years.

In this work, first, we aim to pedagogically introduce these important geometrical tools and review different approaches and key results, with a focus on quantum systems. Secondly, we obtain general principles of optimization of slowly driven quantum thermal machines. These include the constant speed of control variation according to the thermodynamic metric, the absence of quantum coherence, and the optimality of small cycles around the point of a maximal ratio between heat capacity and relaxation time for Carnot engines.

***Entropy* Best Paper Award Committee**

*Entropy* Editorial Board
